# Modulation of α-Synuclein Aggregation In Vitro by a DNA Aptamer

**DOI:** 10.1021/acs.biochem.2c00207

**Published:** 2022-08-22

**Authors:** Claire H. Tran, Ranajay Saha, Celia Blanco, Damayanti Bagchi, Irene A. Chen

**Affiliations:** † Program in Biomolecular Sciences and Engineering, Department of Chemistry and Biochemistry, University of California, Santa Barbara, California 93106, United States; ‡ Department of Chemical and Biomolecular Engineering, University of California, Los Angeles, California 90024, United States

## Abstract

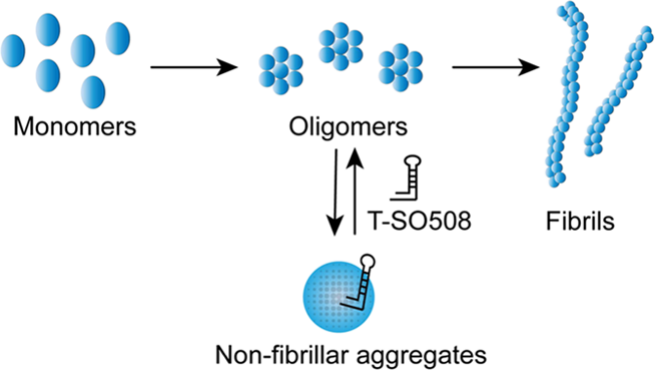

Protein aggregation is an important problem for human health and biotechnology, with consequences in areas ranging from neurodegenerative diseases to protein production yields. Methods to modulate protein aggregation are therefore essential. One suggested method to modulate protein aggregation is the use of nucleic acid aptamers, that is, single-stranded nucleic acids that have been selected to specifically bind to a target. Previous studies in some systems have demonstrated that aptamers may inhibit protein aggregation, including for α-synuclein, a protein implicated in synucleinopathies. However, the mechanisms by which aptamers might affect or modulate aggregation have not been fully determined. In this study, we investigated the effect of an aptamer that binds α-synuclein oligomer, T-SO508, on α-synuclein aggregation in vitro using thioflavin T to monitor aggregation kinetics, and we performed atomic force microscopy, transmission electron microscopy, and analytical ultracentrifugation to characterize intermediate structures. The results indicated that T-SO508, but not control DNA sequences, extends the lag phase of aggregation and stabilizes formation of a small non-fibrillar aggregate complex. Attempts to use the aptamer-induced complexes to seed fibril formation did not in fact accelerate aggregation, indicating that these structures are off-pathway for aggregation. This study highlights a potential mechanism by which aptamers may modulate the aggregation properties of proteins.

## Introduction

Protein aggregation is an issue that underlies several diseases of human health as well as practical problems in biotechnology. While most proteins are typically soluble, some present altered structures and expose aggregation-prone sites in response to stressors, such as a change in the chemical environment. These structurally vulnerable proteins may accumulate together and develop into aggregated forms. Several neurodegenerative diseases, including Parkinson’s disease, are characterized by proteins aggregating to form insoluble fibril structures.^1^ In addition, protein aggregation can be problematic for production and storage of protein biologics, as aggregation negatively affects production yield and can cause unwanted immunogenicity.^2^ Given the serious concerns engendered by protein aggregation, the development and characterization of molecules that might modulate such behavior is desirable.

Aptamers are nucleic acid sequences that bind specifically to a target^3−5^ and are usually discovered through in vitro selection of a randomized pool of DNA or RNA sequences for binding activity. Aptamers are potentially useful for molecular diagnostics^6−8^ and are also considered for therapeutic applications (e.g., pegaptanib sodium, or Macugen).^9^ Compared to antibodies, the benefits of aptamers in such applications are small size, non-immunogenicity, and stability during storage.^10^ Aptamers have been proposed as potential inhibitors for protein aggregation,^11^ and indeed there have been several reports of aptamers exhibiting this capacity.^12−15^ In vitro studies on amyloid β (Aβ) have demonstrated that RNA aptamers inhibit fibrillation of Aβ1–40, despite relatively low binding affinity.^12^ Similar studies on tau protein showed that RNA aptamers against tau prolonged the oligomerization phase of tau.^13^ These in vitro effects can correspond to effects in the cellular setting as well. The aptamers against tau protein reduced the cytotoxic effects of tau overexpression, and also reduced the neurotoxicity of extracellular tau oligomers on primary cell culture neurons.^13^ RNA aptamers selected against monomeric segments of mutant huntingtin (51Q-mhtt and 103Q-mhtt) improved the solubility of mhtt in yeast cells and fixed endocytotic defects caused by aggregation of 103Q-mhtt.^14^ Finally, peptide-mediated delivery of DNA aptamers selected against monomeric α-synuclein into cell lines overexpressing α-synuclein resulted in a reduction in toxicity, recovery of mitochondrial function, and improvements from cellular defects.^15^ These studies established the general phenomenon that aptamers can inhibit protein aggregation, potentially with positive effects at the cellular level.

As seen in the studies discussed above, intrinsically disordered proteins (IDPs) are of special interest for aptamer-based inhibition of aggregation, due to the connection between IDPs and neurodegenerative diseases. α-synuclein is an IDP expressed in the brain and is associated with multiple neurodegenerative diseases, collectively termed synucleinopathies. Like many IDPs, α-synuclein appears to be natively unfolded in solution, and pathological aggregation results in formation of β-sheet amyloid structures (amyloid fibrils). Although still under debate,^16^ α-synuclein oligomers are considered to be the likely toxic species. A previous line of work has demonstrated promising therapeutic effects of DNA aptamers having nanomolar affinity, selected against an immobilized GST fusion to α-synuclein, delivered to primary neurons^15^ as well as in a mouse model of Parkinson’s disease.^17^ While a reduction of fibril formation was observed in vitro using these aptamers, the molecular mechanism of aptamer-induced inhibition was not studied further. In addition, an independent study reported the development of DNA aptamers selected to bind α-synuclein oligomers.^18^ These aptamers were selected to bind to α-synuclein oligomers under two conditions: a gel-shift assay in earlier rounds and a competitive dot-blot assay in later rounds. Some of these aptamers were further characterized to have dissociation constants in the nanomolar range, establishing high binding affinity. Based on these studies, we undertook an in vitro examination with a DNA aptamer in order to investigate the possible effect on the aggregation of α-synuclein.

The mechanisms by which aptamers may affect protein aggregation are likely to vary depending on the specific system. For example, aptamers raised against monomers may act simply through stabilization and solubilization of the monomeric form,^14^ decreasing the driving force and/or rate of aggregation. Here, we focused on a DNA aptamer that had been selected to bind α-synuclein oligomers.^18^ Our investigation suggests that the T-SO508 aptamer modulates α-synuclein aggregation through creation of an alternative non-fibrillar aggregate species. The results broaden the understanding of possible molecular mechanisms underlying aptamer-induced effects on protein aggregation.

## Methods and Materials

### α-Synuclein Expression and Purification

The pt7–7 construct containing α-synuclein (UniProtKB SNCA: P37840) was transformed into *Escherichia coli* BL21(DE3)-competent cells (New England Biolabs). The construct was a gift from A. Buell, namely, pT7–7 asyn WT (Addgene plasmid # 36046, gift of Hilal Lashuel; http://n2t.net/addgene:36046; RRID:Addgene_36046).^19^ α-Synuclein expression was induced at an optical density (OD_600_) of 1–1.2 with 1 mM isopropyl-1-thio-β-d-galactopyranoside (Sigma-Aldrich) at 37 °C for 4–5 h. Cells were harvested at 5000 × *g* for 15 min and resuspended in water. The cell resuspension was lysed at approximately 90 °C for 15 min. Lysate was centrifuged at 30,000 × *g* for 40 min. The supernatant was collected, and protein was precipitated using equal volume 4.5 M ammonium sulfate. The solution was centrifuged at 15,000 × *g* for 30 min. The precipitate was re-suspended in 25 mM Tris–HCl, pH 8.0, and dialyzed against 25 mM Tris–HCl, pH 8.0. α-Synuclein was purified using anion exchange chromatography and size exclusion chromatography. For anion exchange, the protein solution was loaded onto a HiTrap QFF column (GE Healthcare), which was equilibrated to 25 mM Tris–HCl, pH 8.0. The elution gradient was set from 0 to 800 mM NaCl. Fractions containing α-synuclein were identified by SDS-PAGE, collected, and concentrated using Amicon Ultra 15 Centrifugal Filter units (Millipore Sigma). The concentrated protein sample was then loaded on a HiPrep 16/60 Sephacryl S-200 HR column (GE Healthcare). Fractions containing α-synuclein were identified by SDS-PAGE and collected. Samples were flash-frozen in liquid nitrogen and stored in a -80C freezer for subsequent studies. Commercially purchased α-synuclein was obtained from AlexoTech.

### DNA Sequences

DNA sequences used here were based on previously published works^18,20^ (Table 1). The sequences were synthesized and purchased from Bioneer.

**Table 1:** DNA Sequences Used in this Study

name	sequence (5′→3′)
T-SO508 aptamer	GCCTGTGGTGTTGGGGCGGGTGCG
Ran.DNA	GGCGGCTGTGTGGCGGTGTGTCGG
thrombin aptamer	GGTTGGTGTGGTTGG
poly-T sequence	TTTTTTTTTTTTTTTTTTTTTTTT

### α-Synuclein Aggregation Assays Monitored by Fluorescence

Aggregation of α-synuclein was performed in a Tecan M200 Pro instrument. Unless otherwise specified, experiments contained a buffer composition of 140 μM α-synuclein, 10 mM Tris–HCl (pH 7.4), 150 mM sodium chloride, and 5 mM potassium chloride. For fluorescence experiments, 60 μM Thioflavin T (ThT, excitation at 450 nm, emission at 485 nm) was added. Experiments measuring the effect of aptamers used 0–210 μM aptamer. Mixtures (150 μL) were pipetted into a 96-well plate (Corning) and placed into a plate reader (Tecan). The samples were continuously agitated by orbital shaking of 2 mm (∼280 rpm) using a 3 mm glass bead (Fisher Scientific) at 37 °C. Time zero was defined as the start of agitation. Fluorescence measurements were taken every 30 min. To account for background fluorescence and equilibration of the sample, the fluorescence measured at t = 1 h was used as a constant for background subtraction.

### Determination of Lag Time and Growth Rate of α-Synuclein Aggregation by Model-Independent Analysis

We measured the lag phase as defined by Shoffner et al.^21^ A general scheme of the model-independent (MI) method is depicted in Figure S1. Data were smoothed using a moving average to calculate the first derivative. A Gaussian was fit to the first derivative data set to determine the time of the peak value. The peak time was located on the original data set, and the slope of fluorescence increase (growth rate) was calculated. The intersect of the growth rate tangent at the peak time and the average baseline fluorescence value determined the length of lag phase, *t*_lag_.

### Determination of Nucleation and Autocatalytic Growth Rates by the Finke–Watzky Model

The normalized fluorescence data were fitted to the Finke–Watzky (FW) two-step aggregation model.^22,23^
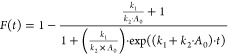
where *F*(*t*) represents the polymeric form of the protein aggregate and *k*_1_ and *k*_2_ correspond to the average rate constants for nucleation and growth. *A*_0_ is the initial concentration of monomeric form of the protein (*A*_0_ = 140 μM).

Fittings were performed using Origin.^24^ Data sets were normalized using the average of the first and last 10 points as minimum and maximum values. Data sets corresponding to the same experiment (e.g., triplicates or sextuplicates) were merged and treated as a single set. Errors in the fitted parameters correspond to the fitting standard error.

We performed three paired *F*-tests to compare aggregation dynamics in the absence and presence of the T-SO508 aptamer at three different concentrations. The null hypothesis is that one curve fits all the data points (i.e., both data sets) and the observed difference is purely due to chance. We fitted a single curve to all the data from both data sets (i.e., with and without aptamer) and obtained one estimate for each of the two parameters in the model (*k*_1_, *k*_2_). The alternative hypothesis is that the curves are distinct, and hence, are ruled by different dynamics. The residual sum of squares and degrees of freedom for the combined set are denoted as SSR_comb_ and d*f*_comb_. For the alternative hypothesis, we fit each data set separately to obtain two distinct curves with two different sets of parameters (*k*_1_, *k*_2_) for each data set (i.e., in the absence and presence of the T-SO508 aptamer). The residual sum of squares and degrees of freedom for each pair of independent fits are RSS_1_, RSS_2_, d*f*_1_, and d*f*_2_. The *F* value is then calculated as
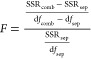
where SSR_sep_ = RSS_1_ + RSS_2_ and d*f*_sep_ = d*f*_1_ + d*f*_2_.

### Seeding of Aggregation

Fresh, filtered α-synuclein (70 μM) was treated using the aggregation assay conditions described above. Reactions either contained no DNA, T-SO508 (45 μM), or thrombin-binding aptamer (70 μM). After 24 h of agitation, samples were collected and centrifuged for 30 min at 16,000 rcf at 4 °C to sediment amyloid fibrils. The supernatant (presumed to contain “seeds” and include oligomers and monomers) was collected, and 2–3 μL of salt-activated nuclease (Sigma Aldrich) was added. The mixtures was incubated at 25 °C for 3 h for digestion of DNA. 10% of the 150 μL volume of the aggregation reaction solution consisted of a seed mixture as well as fresh, filtered α-synuclein (70 μM), buffer, and ThT. Samples were monitored using ThT fluorescence, as described above.

### Atomic Force Microscopy

Samples were imaged on an Asylum MFP-3D Standard System (Asylum Research, Santa Barbara, CA). Prior to imaging, samples were prepared by desalting reaction mixtures using a Zeba Spin Desalting Column, 7 k MWCO (Thermo Scientific). Desalted samples were deposited onto a cleaved mica surface. Imaging was done in AC mode with FORTA probes (AppNano, Santa Clara, CA). Image processing and analysis was performed using Gwyiddion (http://gwyddion.net/).

### Transmission Electron Microscopy

When noted, samples were centrifuged at 14,000*g* for 45 min to remove fibrils for improved contrast of smaller aggregates. Supernatant or whole solution samples (5 μL) were deposited to a previously discharged carbon-coated 300 mesh copper grid (Ted Pella, Inc.) and allowed to sit for 5 min. The excess liquid was washed with water and removed with filter paper. Grids were then negatively stained for 2 min with 2% uranyl acetate, and excess liquid was removed with filter paper and again washed. The negatively stained samples were dried in air and imaged on an FEI Tecnai T12 transmission electron microscope operated at 120 kV. Images of the total solution (without centrifugation) were also obtained by the same method.

### Analytical Ultracentrifugation

Sedimentation velocity experiments were performed at 20 °C in a Beckman Optima XL-A analytical ultracentrifuge using absorption optics at 260 nm. A 12 mm pathlength double-sector cell was used. Fresh, filtered α-synuclein (140 μM) in the presence of specific DNAs (T-SO508 or Ran.DNA, 40 μM each) were treated using the aggregation assay conditions described above. Diluted samples (O.D._260_ < 1) were run at 55,000 rpm. A partial specific volume of 0.733 for α-synuclein, calculated from the amino acid composition and corrected to 20 °C,^25,26^ was used. Partial specific volumes of 0.55 for T-SO508 and 0.670 for the 1 to 1 complex were used. Apparent sedimentation coefficient distributions, uncorrected for diffusion, were determined as *g*(*s*) plots using the Beckman Origin-based software (Version 3.01). These plots display a function proportional to the weight fraction of material with a given sedimentation coefficient, *s*. The function *g*(*s*) was calculated as *g*(*s*) = (d*c*/d*t*)(1/*c*_o_)(ω^2^*t*^2^/ln(*r*_m_/*r*))(*r*^2^/*r*_m_^2^), where *s* is the sedimentation coefficient, ω is the angular velocity of the rotor, *c*_o_ is the initial concentration, *r* is the radius, *r*_m_ is the radius of the meniscus, and *t* is time. The *x*-axis is converted to the sedimentation coefficient by *s* = (1/*ω*^2^*t*)(ln(*r*/*r*_m_)). These plots display a function proportional to the weight fraction of material with a given sedimentation coefficient, *S*.^27^

Theoretical sedimentation coefficients were calculated using the Svedberg equation using an *f*/*f*_o_ of 1.3.

## Results

### Aptamer T-SO508 Perturbs α-Synuclein Aggregation

Aggregation of α-synuclein was assayed over time by ThT fluorescence. ThT is a cationic dye whose fluorescence increases upon interaction with protein fibrils, allowing for kinetic monitoring of amyloid formation.^28^ Aptamer T-SO508 was previously reported to bind to α-synuclein oligomers with a dissociation constant (*K*_D_) of 68 nM.^18^ To probe the effect of T-SO508 on α-synuclein aggregation, T-SO508 was added in varying concentrations to 140 μM α-synuclein. Aggregation was induced through agitation of glass beads in a 96-well plate at 37 °C and monitored in a fluorescence plate reader.

**Figure 1:**
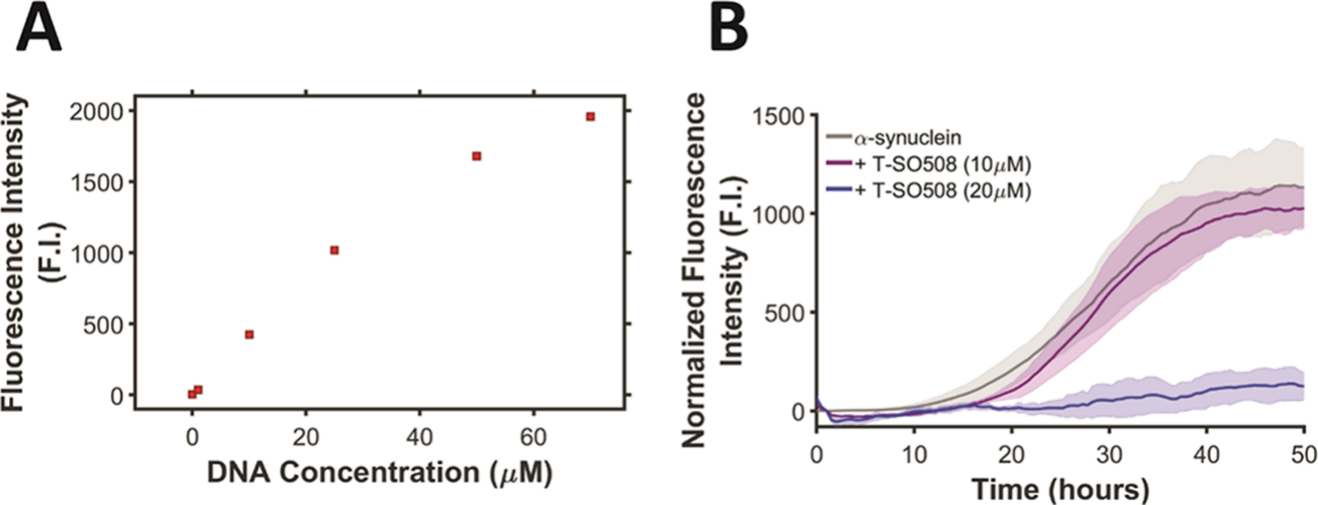
α-Synuclein aggregation in the presence and absence of T-SO508, monitored by thioflavin T (ThT) fluorescence. (A) Dependence of fluorescence on the T-SO508 aptamer concentration without α-synuclein (60 μM ThT). (B) α-Synuclein (140 μM) aggregation in the presence of 60 μM ThT and 0 (gray), 10 μM (purple), or 20 μM (blue) T-SO508. The shaded area represents 1 standard deviation (sample size, *n* = 3–5). The fluorescence intensity at 1 h was taken as the background level for baseline correction (F.I. = fluorescence intensity units).

In the absence of the aptamer, α-synuclein aggregation was observed as a sigmoidal rise in fluorescence over approximately 2–3 days. We noted that the aptamer itself (without α-synuclein) caused increased background fluorescence, consistent with the known interaction between ThT and DNA structures.^29,30^ ThT fluorescence increased linearly with the concentration of T-SO508 up to a T-SO508 concentration of roughly 30 μM (Figure 1A). Given this background, baseline correction of the fluorescence data was performed by subtraction of the background fluorescence intensity. Little change in ThT fluorescence was observed for α-synuclein samples in the presence of 20 μM T-SO508 (0.14 equivalents), suggesting that this aptamer modified kinetics of aggregation (Figure 1B). In contrast, adding a negative control DNA sequence with the same base composition as T-SO508 (Ran.DNA, 40 μM) gave no apparent effect on the aggregation kinetics of α-synuclein (Figure S2).

The negligible increase in fluorescence at higher T-SO508 concentration (20 μM) could be interpreted as a relative lack of fibril formation. However, another possible explanation was considered, specifically that it was possible that the aptamer DNA bound all of the ThT, leaving no ThT available to bind α-synuclein fibrils if present. In this case, α-synuclein fibrils might form, but because ThT was not available to bind the fibrils, the fibrils would not be detected as a fluorescence increase. To test whether fibrils could be detected by ThT fluorescence increase in the presence of T-SO508, aptamer (20 μM) and ThT (60 μM) were pre-mixed to allow aptamer and ThT to bind each other, giving the background fluorescence of the solution. Separately, preformed fibrils were prepared by incubation of α-synuclein for 90 h under aggregation conditions. The preformed fibrils were then added to the solution of ThT and T-SO508 to determine whether the addition of fibrils would result in an increase in ThT fluorescence (Figure S3). Indeed, the absolute fluorescence of the sample increased by 12% after the addition of preformed fibrils, compared to a control (Table S1). The significant fluorescence increase upon addition of fibrils indicates that the concentration of the aptamer used in this experiment did not saturate the binding interactions of ThT, such that newly formed amyloid fibrils would still be detectable. These results support the use of these conditions (buffer, ThT and aptamer concentration) to monitor aggregation kinetics and fibril growth in the presence of T-SO508. Therefore, the lack of sigmoidal fluorescence increase observed for α-synuclein in the presence of 20 μM T-SO508 under aggregation conditions is suggestive of perturbation of protein aggregation by T-SO508.

### Effect of Low Concentrations of Aptamer T-SO508 on α-Synuclein Aggregation Kinetics Monitored by ThT Fluorescence

While qualitatively interesting, the results in the presence of high concentrations (≥20 μM) of T-SO508 were difficult to interpret quantitatively since little or no dynamics were observed. Therefore, we examined the effect of low concentrations of T-SO508 on α-synuclein aggregation. Low concentrations of T-SO508 (≤10 μM) allowed for observation and analysis of kinetics of α-synuclein aggregation, at 140 μM α-synuclein, using ThT (Figure 1B). We used two methods to quantify the effect of these low T-SO508 concentrations on aggregation kinetics: an MI, phenomenological analysis as well as fitting to the two-step FW model of aggregation.

**Figure 2:**
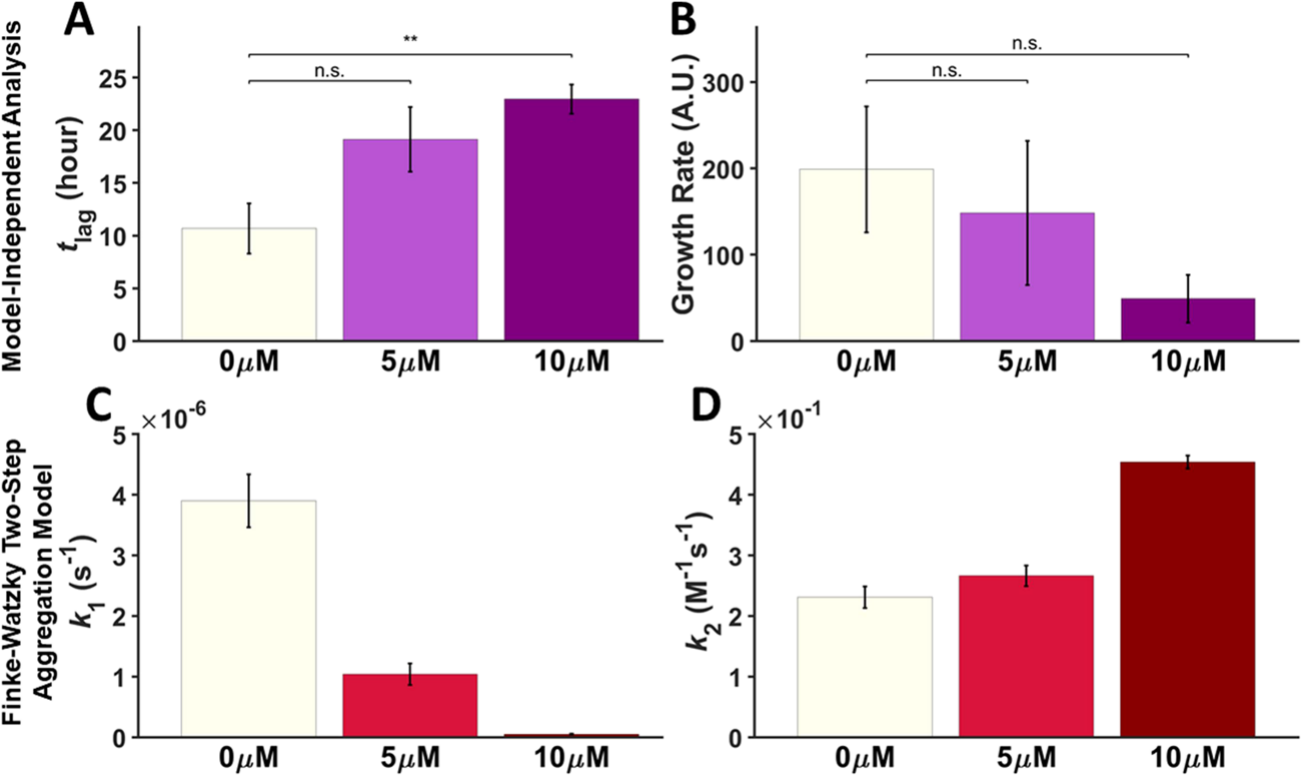
Effect of the T-SO508 aptamer on aggregation kinetics. Samples contained 140 μM α-synuclein, 60 μM ThT, and 0, 5, or 10 μM T-SO508 (as indicated). Fluorescence traces were analyzed by MI analysis (A,B) or by the FW model (C,D). Error bars for A and B represent one standard error (*n* = 6). MI analysis confirmed the slowed initial steps in the presence of T-SO508, seen by an increase in *t*_lag_ (A) and little or no effect on growth rate (B). ** indicates *p* < 0.01; n.s. = not significant. For FW analysis, the global fit of *k*_1_ and *k*_2_ demonstrated a pronounced decrease in the nucleation rate (*k*_1_; part C) in the presence of the T-SO508 aptamer and a slight increase in the growth rate (*k*_2_; part D). Error bars for C and D represent the fitted parameter standard error for each condition.

In the MI approach, the sigmoidal aggregation curve is characterized by a lag time and a growth rate. The lag time (*t*_lag_) was defined by Shoffner et al.^21^ as the time between the start of the experiment and the time of the intersection between the tangent drawn at the point of maximum growth rate and the average baseline fluorescence value (Figure S1). At 10 μM T-SO508 aptamer, *t*_lag_ was increased significantly compared to no aptamer (Figure 2A). In MI analysis, the growth rate is measured as the slope of the tangent line at the highest rate of change, the position of which is determined by the maximum of the first derivative of the data set. No significant change was observed in the growth rate when the T-SO508 aptamer was added (Figure 2B).

The FW aggregation model^22,23^ is based on two elementary kinetic steps. The first step models conversion of the protein to an aggregation-prone state (i.e., nucleation), and the second step models autocatalytic conversion to the aggregated state (i.e., growth). Data are fit to this model with two parameters, namely, the rate of nucleation (*k*_1_) and the autocatalytic growth rate (*k*_2_) (Figure S4). Upon fitting the data, the presence of the T-SO508 aptamer resulted in a substantial decrease in the nucleation rate (by ∼4-fold at 5 μM T-SO508 and ∼75-fold at 10 μM T-SO508) (Figure 2C), consistent with the observation of prolonged *t*_lag_ in the MI analysis. A slight increase in the autocatalytic growth rate was also observed (∼2-fold at 10 μM T-SO508; Figure 2D). The small size of the effect on *k*_2_ is consistent with the lack of statistically significant effect seen in MI analysis of the growth rate.

To further assess the statistical significance of the differences seen, an *F*-test^31^ was used to determine whether the aggregation dynamics were different in the absence vs presence of the T-SO508 aptamer (5 and 10 μM). For the *F*-test, the different data sets (aggregation in the absence vs presence of the aptamer) were fitted together using the FW model, under the assumption that they follow the same dynamics, resulting in a single set of fitted parameters (*k*_1_ and *k*_2_). Then, the data sets were fitted separately, assuming that the aptamer does affect kinetics, resulting in two independent sets of *k*_1_ and *k*_2_. Finally, the fittings were globally compared using an *F*-test (see Methods), giving *F*-values of 136 and 812 for the T-SO508 aptamer concentrations of 5 and 10 μM, respectively (*p* < 10^–5^ in both cases), supporting the conclusion that the T-SO508 aptamer significantly changes aggregation dynamics. In further analyses described below, the MI approach was preferred since the results were concordant between MI and FW analysis, and the appropriateness of the FW model for reactions containing aptamer was uncertain.

**Figure 3:**
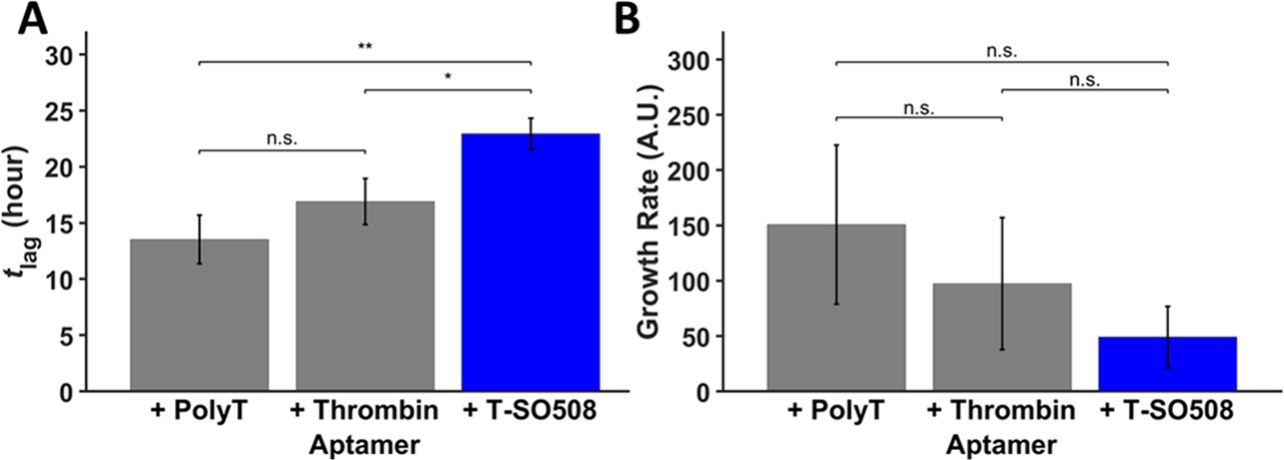
Comparison of effects of the T-SO508 aptamer and control DNA sequences. Samples contained 140 μM α-synuclein, 60 μM ThT, and 10 μM of one of the following: poly-T sequence, thrombin aptamer, or T-SO508. Fluorescence traces were analyzed by MI analysis. (A) T-SO508 prolonged *t*_lag_ significantly compared to control sequences. (B) In contrast, the growth rate shows no significant difference between T-SO508 and control sequences. Error bars represent one standard error (*n* = 6; two-sample *t* test: n.s. = not significant; * indicates *p* < 0.05; ** indicates *p* < 0.01).

To determine whether the effects observed were specific to T-SO508, two control DNA sequences (a thrombin-binding DNA aptamer^20^ and poly-T 24-mer sequence) were studied in addition to Ran.DNA (Figure S2) and incubated with α-synuclein under the same conditions. As expected, the MI analysis showed that *t*_lag_ in the presence of T-SO508 was significantly longer than *t*_lag_ in the presence of the control sequences (Figure 3A). These findings indicated that initial steps in the aggregation process were perturbed specifically by T-SO508. Consistent with the results in the absence of aptamer, for the growth rate, little or no difference was observed between T-SO508 and the control sequences (Figure 3B).

### Characterization of Small Aggregated Structures Formed in the Presence of Aptamer T-SO508

**Figure 4:**
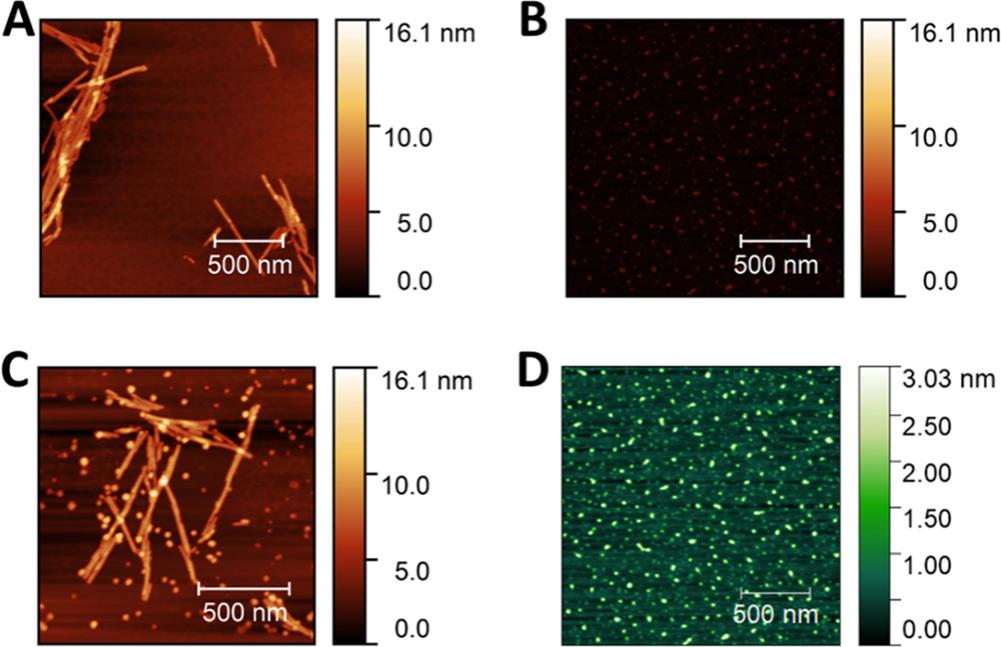
Observation of small non-fibrillar aggregates induced by T-SO508 aptamer using AFM. All samples were desalted, diluted (1:100), and deposited onto freshly cleaved mica. AFM height is shown by the heat map as indicated by the scale bars. (A) α-synuclein (70 μM) after 96 h of aggregation; (B) T-SO508 (210 μM) aptamer deposited onto mica; (C) α-synuclein (70 μM) with the T-SO508 aptamer (210 μM) after 96 h of aggregation conditions; (D) same as (B) but with the height scale adjusted for improved contrast.

Samples of α-synuclein incubated with high concentrations of T-SO508 were studied by atomic force microscopy (AFM) to understand any morphological changes of the particles. AFM was used to image the aggregates that developed in samples with or without excess T-SO508 (70 μM α-synuclein with or without 210 μM T-SO508). Consistent with the studies mentioned above, a ThT kinetic assay suggested perturbed aggregation in the presence of 210 μM T-SO508 (Figure S5). In the absence of T-SO508, α-synuclein aggregation produced fibrils, as expected, after ∼96 h (Figure 4A). T-SO508 by itself was detectable by AFM as objects roughly 1.9 nm in height (Figure 4B,D), consistent with the visibility of similarly sized single-stranded nucleic acids using AFM.^32^ In contrast, in the presence of excess T-SO508 (molar ratio 3:1), α-synuclein subjected to aggregation conditions for ∼96 h resulted in many roughly spherical, non-fibrillar structures (Figure 4C), in addition to fibrils. The size of the spherical structures (∼7.4 nm in height) was significantly larger than T-SO508 itself (∼1.9 nm in height) (Figures S6–S7), indicating that the structures observed were not simply deposited DNA. The observation of these structures suggests that T-SO508 induced formation of small, non-fibrillar aggregates with α-synuclein, which persisted for an extended period of time, that is, beyond the point after which fibril formation of a sample lacking T-SO508 would have been complete.

**Figure 5:**
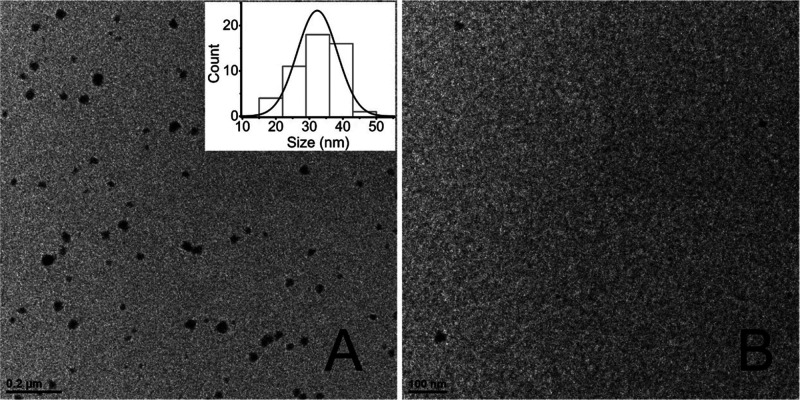
TEM observation of spherical aggregates in the presence of the T-SO508 aptamer. Samples were centrifuged to pellet fibrils, and the supernatants were collected and negatively stained by uranyl acetate for imaging. (A) α-synuclein (140 μM) with the T-SO508 (40 μM) aptamer, with size distribution shown. (B) α-synuclein (140 μM) with Ran.DNA (40 μM). See Figure S2 for ThT assay results under these conditions. Additional TEM images from different grid positions are shown in Figures S8 and S9.

To further examine the non-fibrillar aggregates, α-synuclein samples containing either T-SO508 or Ran.DNA (40 μM of either) were incubated for 72 h and then centrifuged to remove fibrils. Transmission electron microscopy (TEM) imaging was performed using the supernatant solutions. ThT fluorescence under these conditions had shown a lack of detectable increase in the presence of T-SO508, but a sigmoidal increase, typical of unperturbed α-synuclein aggregation, in the presence of Ran.DNA (Figure S2). Consistent with AFM, spherical, non-fibrillar aggregates were detected with α-synuclein, with size centered around 32 nm (Figure 5A, inset; Figure S8) in the presence of T-SO508 by TEM. In contrast, such structures were absent with α-synuclein in the presence of Ran.DNA (Figures 5B and S9). Samples imaged by TEM without centrifugation to remove fibrils confirmed that the non-fibrillar aggregates were observed when α-synuclein was incubated in the presence of T-SO508 but not Ran.DNA (Figure S10).

**Figure 6:**
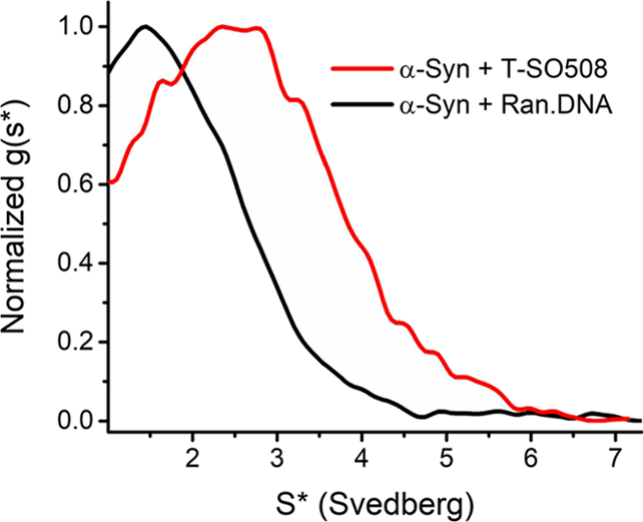
Normalized sedimentation coefficient distribution (uncorrected for diffusion) of α-synuclein samples incubated for 72 h, with either T-SO508 (red) or Ran.DNA (black), measured by AUC (sedimentation velocity of 55,000 rpm at 20 °C).

These samples were further characterized by analytical ultracentrifugation (AUC) to compare the sedimentation of structures found in the solution of α-synuclein with T-SO508 or Ran.DNA. Absorbance at 260 nm was used for detection, which reports primarily on the DNA since α-synuclein lacks tryptophan residues (Figure S11). In addition, the ultracentrifugation speed rapidly pellets large aggregates, such as fibrils and larger oligomers, so that smaller particles are observed in this technique. The apparent sedimentation coefficient distribution (*g*(s*)) of the sample with Ran.DNA was centered at 1.45 Svedberg units (S) (Figure 6). However, the sample with T-SO508 showed a broader peak width centered at 2.55 S (Figure 6), indicating a substantially larger complex. The observation of larger complexes with T-SO508 compared to Ran.DNA is consistent with the TEM and AFM studies, indicating that T-SO508 results in formation of alternative globular structures. In addition, although the absorbance spectrum is broad and contribution of α-synuclein is not large (Figure S11), the DNA:protein ratio can be approximately assessed by the *A*_260_/*A*_276_ ratio (Table S2). This comparison shows that, after aggregation, the supernatant of the T-SO508 sample has a significantly smaller DNA:protein ratio than the Ran.DNA sample supernatant, consistent with reduced protein aggregation in the T-SO508 sample compared to the Ran.DNA sample.

### Aggregates Formed with T-SO508 Do Not Seed Fibril Formation

Given that the presence of T-SO508 prompted formation of small aggregates, presumed to be complexes containing protein and DNA, we sought to determine whether these mixed structures were competent to act as seeds to form fibrils (i.e., accelerating aggregation kinetics by reducing the lag time). We prepared putative oligomers by 24-hour incubation of α-synuclein (70 μM) under aggregation conditions, in the presence or absence of T-SO508 (45 μM). This intermediate concentration of T-SO508 was chosen to avoid excessive unbound DNA while also showing perturbation of aggregation kinetics (Figure S12). Fibrils were removed by centrifugation, and excess DNA was degraded by DNase. The remainder was taken as “seeds” for attempted nucleation of aggregation in fresh α-synuclein. “Seeds” made in the presence of the aptamer were compared to seeds made without the aptamer. Seeds were also prepared in the presence of a control sequence (70 μM thrombin-binding aptamer). Kinetics of seeded aggregation were followed by the ThT assay and analyzed by the MI method to determine the effect on seeding behavior, as reflected in *t*_lag_.

**Figure 7:**
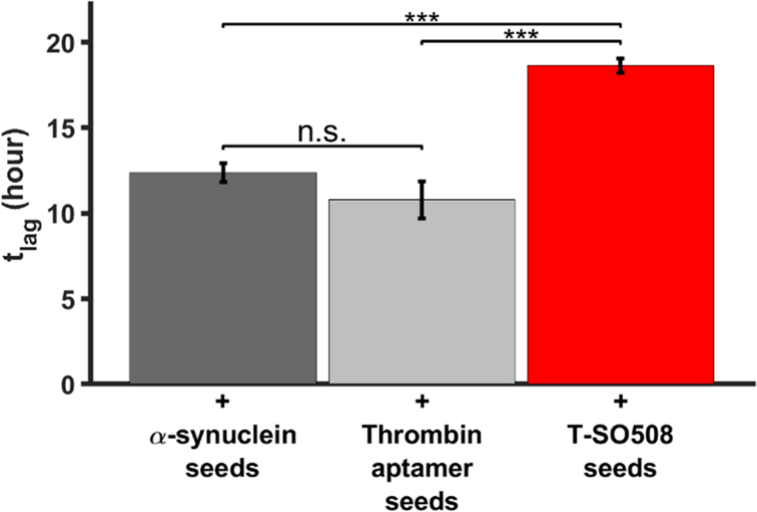
Nonfibrillar aggregates formed in the presence of the T-SO508 aptamer do not seed aggregation. Seeds were formed with 70 μM α-synuclein and one of the following: no DNA (“α-synuclein seeds”; dark gray), 70 μM thrombin aptamer (“thrombin aptamer seeds”; light gray), or 45 μM T-SO508 aptamer (“T-SO508 seeds”; red). Seeds recovered after DNase treatment were then added to a fresh solution of α-synuclein (140 μM). ThT fluorescence was used to monitor aggregation. Data were analyzed by the MI method. Seeds developed in the presence of T-SO508 do not shorten *t*_lag_ (T-SO508 seeds), in contrast to seeds developed with the control DNA or no DNA (α-synuclein seeds and thrombin aptamer seeds). Error bars are one standard error (*n* = 4; *p*-values for the two-sample *t*-test are indicated: n.s. = not significant, *** indicates *p* < 0.001).

The lag time using “seeds” developed in the presence of T-SO508 was significantly longer than that with seeds developed without DNA and seeds developed in the presence of the thrombin-binding aptamer (Figure 7). Indeed, the lag time using “seeds” developed with T-SO508 was statistically indistinguishable from that of an unseeded experiment, although the variation in lag times in unseeded experiments was larger (Figure S13). These results indicate that T-SO508-induced structures do not promote aggregation, in contrast to standard α-synuclein oligomers. On the other hand, the presence of the thrombin-binding aptamer did not affect the seeds’ ability to promote aggregation (*t*_lag_ statistically indistinguishable with and without the thrombin-binding aptamer; Figure 7), indicating an effect specific to T-SO508. These results indicate that structures formed with T-SO508 are not competent for accelerating aggregation and are biophysically distinct from pure α-synuclein seeds.

## Discussion

In this study, we investigated the effect of a DNA aptamer in modulating α-synuclein protein aggregation. We focused on T-SO508, which had been previously selected and demonstrated to bind α-synuclein oligomers with ∼68 nM affinity.^18^ Here, the assays were performed with micromolar concentrations, so it can be assumed that nearly all of the aptamer was bound in these studies. The initial studies on aggregation kinetics, using established ThT fluorescence assays, suggested that relatively low levels of aptamer (20 μM aptamer with 140 μM α-synuclein) may result in perturbed aggregation kinetics. However, these studies were complicated by the known interaction of ThT binding to DNA,^29,30^ which contributed high background fluorescence. While this did not prevent detection of fibrils (Figure 1B), it might have complicated kinetic analysis. Thus, we limited our analysis of kinetic studies to low concentrations of the DNA aptamer. The kinetic analysis showed little or no effect of the aptamer on the growth rate, which reflects the fibril elongation process. In contrast, low levels of aptamer significantly prolonged the lag phase, which reflects nucleation processes. Aggregation kinetics of the protein were unaffected by the presence of control DNA sequences, including one with identical base composition as the aptamer (Ran.DNA). The effect on nucleation processes is consistent with the original selection of the aptamer to target oligomeric species.^18^

Two methods were used to analyze α-synuclein aggregation kinetics: an MI approach and the FW two-step aggregation model. The MI analysis allowed for a phenomenological characterization of an initial lag phase (*t*_lag_) as well as the growth phase of aggregation, independent of a detailed mechanism. The FW model, while simple in having only two parameters, may not correctly represent the mechanism, as it does not treat the initial process of primary nucleation or the generation of intermediates^33,34^ and is not likely to be appropriate for experiments in which aggregation is modified by aptamers. However, more sophisticated reaction schemes that have been proposed are intricate and involve a large number of parameters.^33^ Thus, the FW fitting may also be considered to be essentially phenomenological as applied to these experiments. For cases in which both MI and FW analyses were applied, the results agreed, but we relied primarily on MI analysis here.

To further probe the effect of larger amounts of the aptamer at a structural level, we analyzed the reactions by AFM. These results showed that T-SO508 caused formation of small, roughly round aggregates, which were not seen in α-synuclein alone and also differed from the structures observed in T-SO508 alone. Formation of these small non-fibrillar aggregates in the presence of T-SO508, but not Ran.DNA, was also confirmed by TEM and AUC studies. Interestingly, DNA can promote the formation of protein oligomers through DNA–oligomer networks,^35^ consistent with the observation that T-SO508 led to the development of mixed aggregates with α-synuclein. To establish whether these aggregates represented on-pathway intermediates of aggregation vs off-pathway species, we attempted to seed fresh α-synuclein aggregation reactions with the aptamer-induced aggregates. We found that the aptamer-induced aggregates did not act functionally as nucleation seeds, indicating that the observed aggregates are an off-pathway species. Furthermore, the observation that reactions treated with the aptamer did not form effective “seeds” suggests that they not only developed off-pathway aggregates, but also failed to develop substantial on-pathway oligomeric seeds.

Control experiments with DNA sequences that did not bind α-synuclein demonstrated that the observed effects were specific to T-SO508. Although we do not know the detailed molecular mechanism, modulation of aggregation is reasonable on electrostatic grounds. The net charge of α-synuclein is −9, and association with the DNA aptamer is expected to result in a more negative charge, leading to increased electrostatic repulsion between subunits that might perturb aggregation. Indeed, in a prior study, binding of a DNA aptamer to the N- and C-terminus of monomeric α-synuclein caused inhibition of aggregation due to blockage of long-range interactions within the protein.^15^ In addition, fibrils were observed concomitantly with the alternative aggregates. Since fibril formation is essentially irreversible, fibrils may accumulate even if protein is initially shunted toward an alternative pathway, if the aptamer–protein interactions are transient. Several classes of small molecules, including both polyphenols and non-polyphenolic natural products, have been previously observed to inhibit α-synuclein filament assembly, with IC_50_ values in the low micromolar range.^36−38^ Noncovalent binding between these inhibitors and α-synuclein is based on hydrophobic interactions, aromatic interactions, hydrogen bonding, as well as electrostatic interactions; thus, a variety of interaction types may play a role in the effect of T-SO508 on α-synuclein.

The T-SO508 aptamer was reported to bind α-synuclein oligomers with a *K*_D_ of 68 nM,^18^ but fibrils are still observed at higher aptamer concentrations (e.g., 10 μM). Several factors may contribute to this observation. First, the oligomers used to raise the aptamer had been generated through two cycles of freeze-drying, followed by purification using size exclusion chromatography, but it is unknown how well the aptamer binds to the variety of heterogeneous oligomeric structures during ongoing aggregation. Second, in experiments in which the concentrations of the aptamer and protein were higher than the *K*_D_, the protein was still present in high stoichiometric excess (140 μM in the ThT assays). Third, since aggregation is essentially irreversible, aggregates may still accumulate if the aptamer–protein interactions are transient and in dynamic equilibrium during the experiment.

## Conclusions

Taken together, these results suggested that aptamer T-SO508 modifies α-synuclein aggregation through formation of an off-pathway nonfibrillar species. Given that T-SO508 primarily binds to α-synuclein oligomers, it is likely that these alternative aggregates are formed after association of the aptamer to the protein. Since the alternative aggregates do not promote fibril formation, they are most likely to be off-pathway (Figure S14). To our knowledge, this mechanism has not been previously reported as an effect of an aptamer on protein aggregation. It should be noted that the presence of this mechanism does not necessarily exclude other mechanisms. Given the interest in using aptamers to control or modulate protein aggregation in therapeutic and/or biotechnological settings, the findings highlight a mechanism by which aptamers may influence aggregation properties of their targets.

## Supporting Information Available

ThT assays and data fitting, AFM analysis, TEM images, absorbance ratios, and mechanistic scheme (PDF)

## Accession Codes

SNCA: P37840.
