# Supplementary material for: Modulation of α-Synuclein Aggregation In Vitro by a DNA Aptamer
Source: Biochemistry. 2022 Aug 22;61(17):1757–65. doi: 10.1021/acs.biochem.2c00207 (PMC9454088; doi:10.1021/acs.biochem.2c00207)
Supplement: Supplementary file 1 — bi2c00207_si_001.pdf [file bi2c00207_si_001.pdf]

Supplementary Information

Modulation of α-synuclein aggregation *in vitro* by a DNA aptamer

Claire H. Tran[1^], Ranajay Saha[2^], Celia Blanco[2], Damayanti Bagchi[2], Irene A. Chen[1,2]*

[1] Program in Biomolecular Sciences and Engineering, Department of Chemistry and Biochemistry, University of California, Santa Barbara, CA 93106, USA.

[2] Department of Chemical and Biomolecular Engineering, University of California, Los Angeles, CA 90024, USA

^equal contribution

*Correspondence: ireneachen@ucla.edu

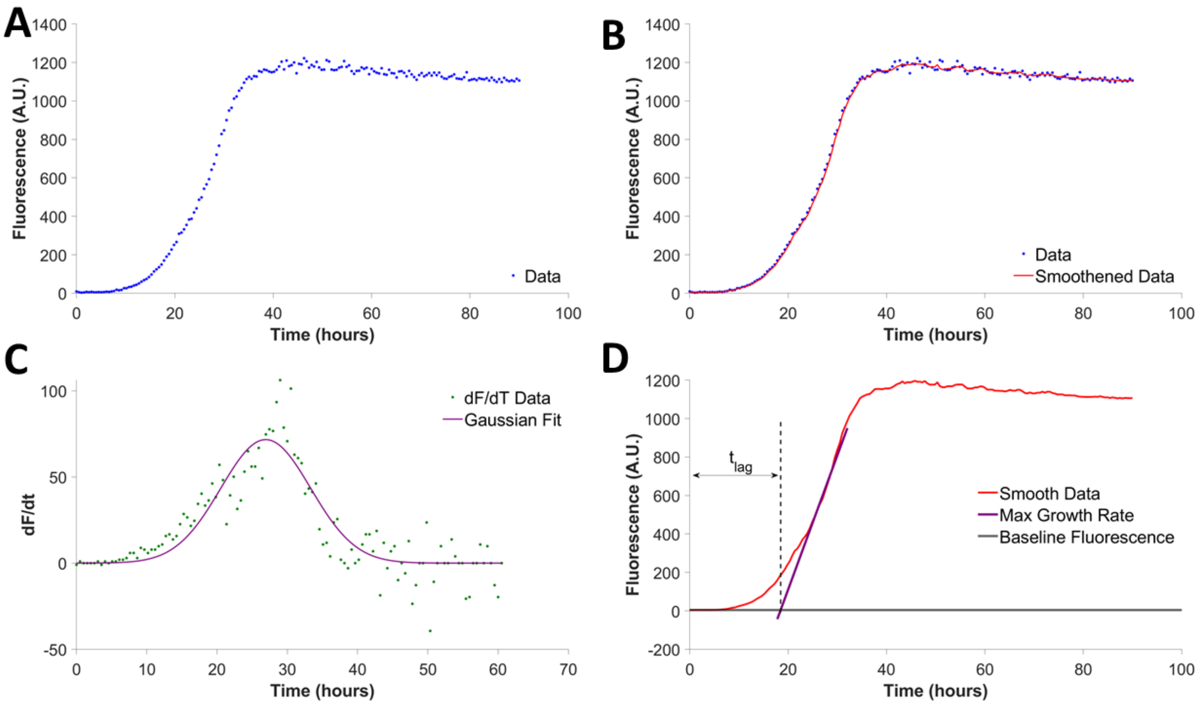

Figure S1. Illustration of model-independent (MI) analysis, conducted individually on each data set (*n*=6) for each condition (9 separate conditions). (A) Fluorescence of Thioflavin T was measured over time (blue). (B) Data sets were smoothed using a moving average (red). (C) The first derivative was calculated on the smoothed data (green) and fit to a Gaussian curve (purple), the center of which was taken as the peak time. (D) To determine t_{lag}, the intercept between the growth rate tangent at the peak time (purple line) and the average of the starting fluorescence (gray line) was calculated. The *x*-coordinate of the intercept is the t_{lag} value (dotted line).

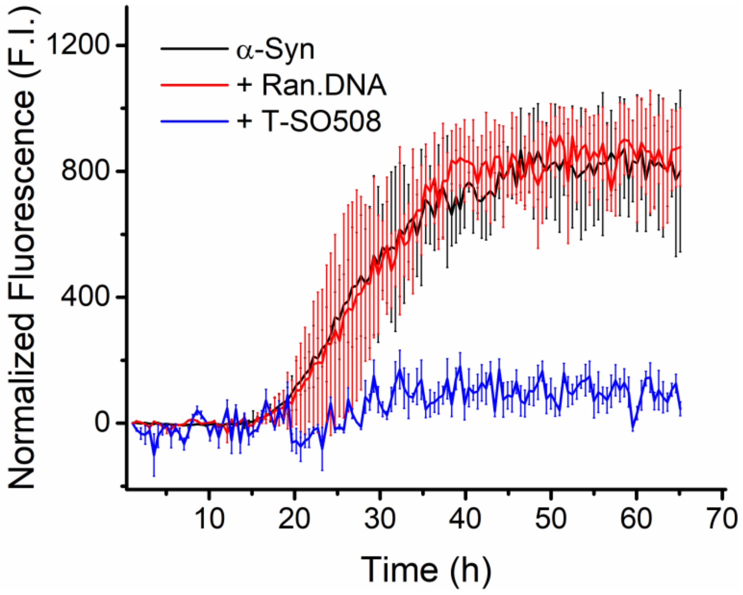

Figure S2. α-synuclein (140 μM) aggregation in the presence of ThT, without DNA (black), in the presence of 40 μM of a randomized control DNA sequence (Ran.DNA, red), or in the presence of 40 μM T-SO508 (blue). Vertical bars represent standard deviation (n = 3). The fluorescence value at 1 hr was subtracted from the data values as background (F.I. = fluorescence intensity units). Protein source: AlexoTech.

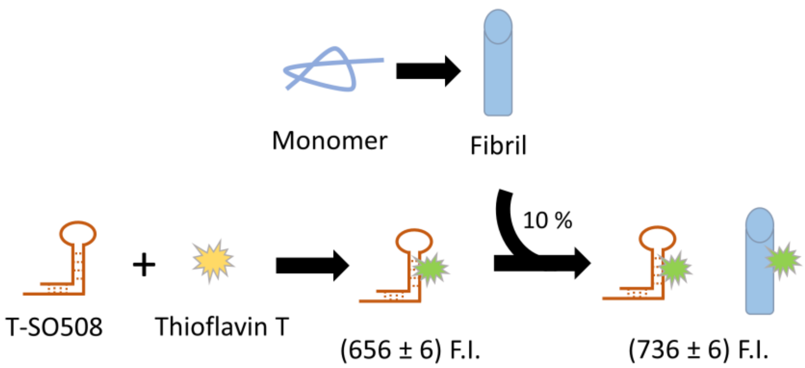

Figure S3. Illustration of experiment confirming the ability of ThT to detect fibrils in the presence of T-SO508.

Table S1. Fluorescence of samples in the presence of 60 μM ThT

Sample	Absolute ThT Fluorescence Intensity (F.I.; relative units)
T-SO508 (20 μM) control (10% volume of water added)	656 ± 6
T-SO508 (20 μM) + Preformed Fibrils (10% volume of preformed fibrils added)	736 ± 6
Fibrils alone	150 ± 3
Buffer	3 ± 1

Figure S4. Data fitting to the Finke-Watzky two-step aggregation model, in the presence of T-SO508 aptamer in the concentrations specified (0 μM, 5 μM, and 10 μM). The gray dots represent normalized data taken at each condition (*n*=6), while the black solid line represents the fitted data from all data collected at the specified condition.

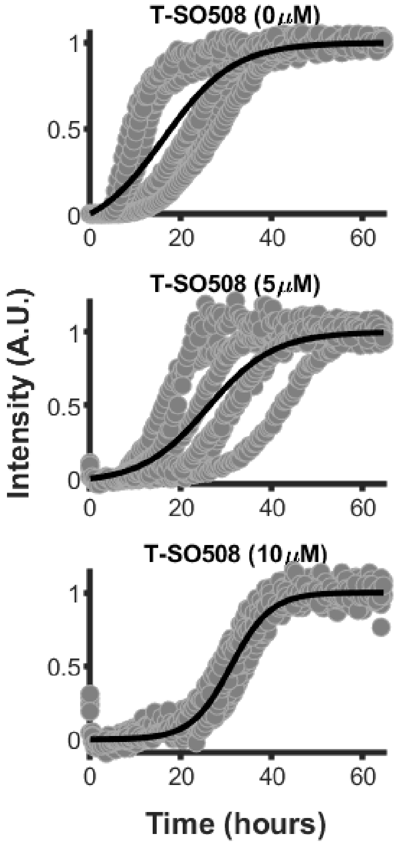

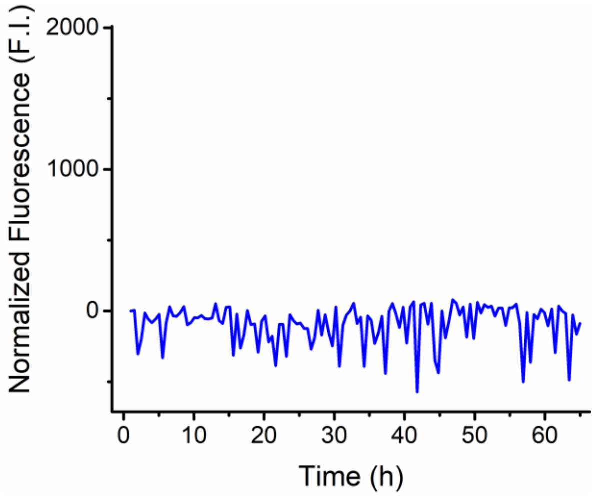

Figure S5. α-synuclein (70 μM) monitored by ThT in the presence of 210 μM T-SO508.

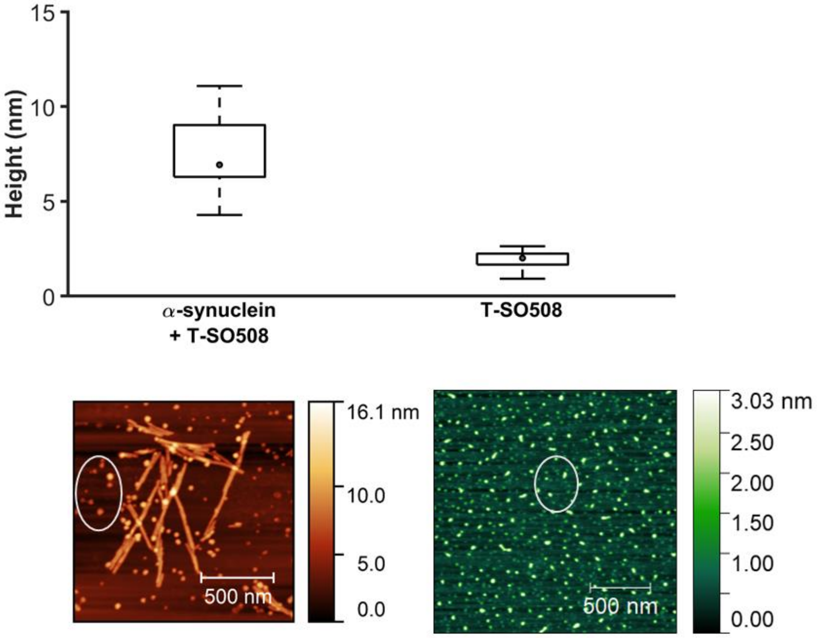

Figure S6. Comparison of height profiles of the spherical shapes (examples are outlined by the white circle) found in samples with α-synuclein and T-SO508 (left) compared to T-SO508 alone (right), determined from AFM images (Figure 4). Error bars represent one standard error (*n*=57 and n=118 for α-synuclein with T-SO508 and T-SO508 alone, respectively). The comparison indicates that the structures formed by α-synuclein and T-SO508 together are distinct from those formed with T-SO508 alone.

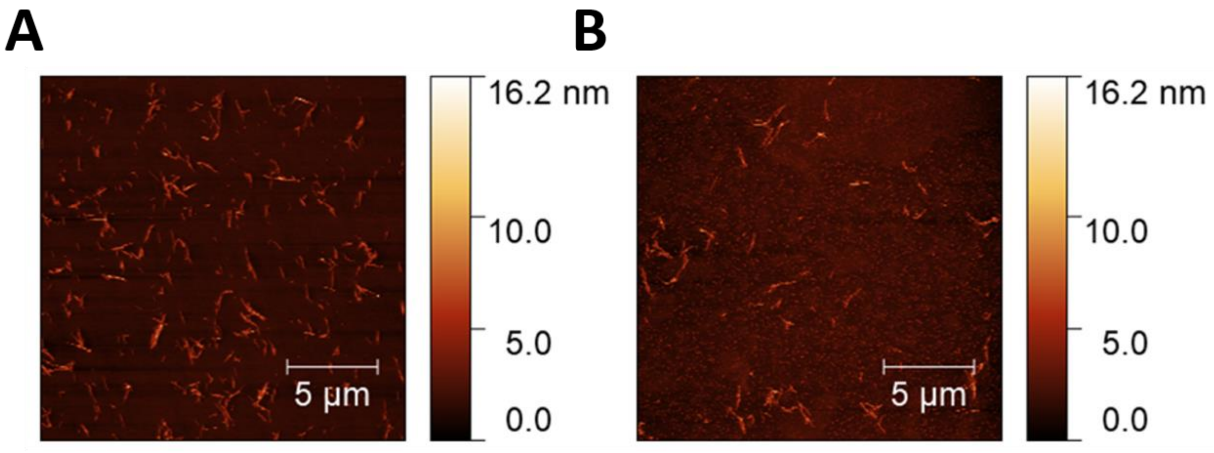

Figure S7. Low-magnification AFM images of (A) α-synuclein (70 µM) after 96 hours of aggregation conditions, and (B) α-synuclein (70 µM) with T-SO508 aptamer (210 µM) after 96 hours of aggregation conditions. A manual count shows 98 fibrils without aptamer and 30 fibrils in the presence of T-SO508.

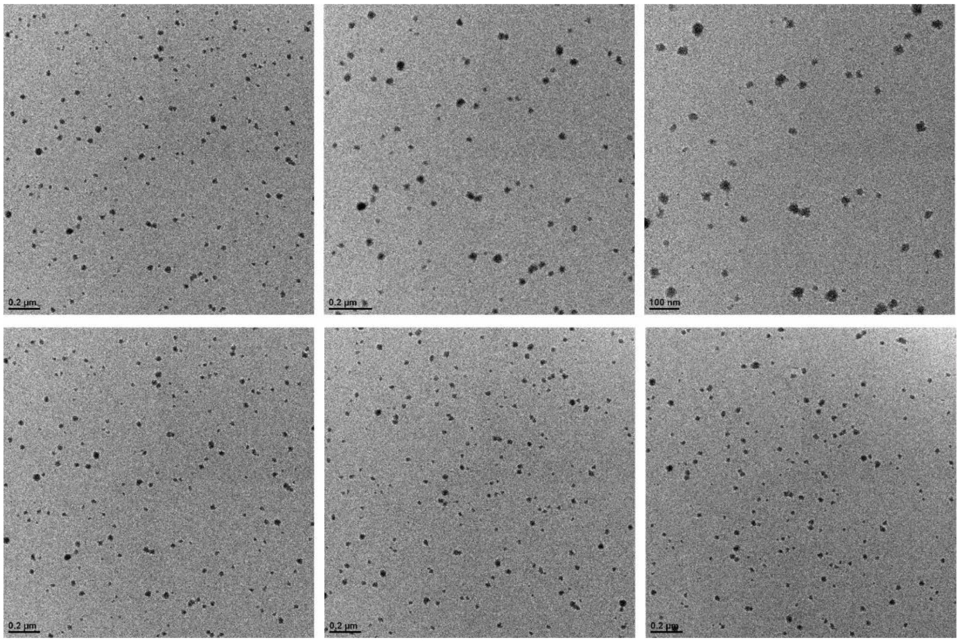

Figure S8. TEM imaging of α-synuclein (140 μM) with T-SO508 (40 μM) aptamer (supernatant after centrifugation), from multiple positions on the TEM grid. Also see Figure 5A.

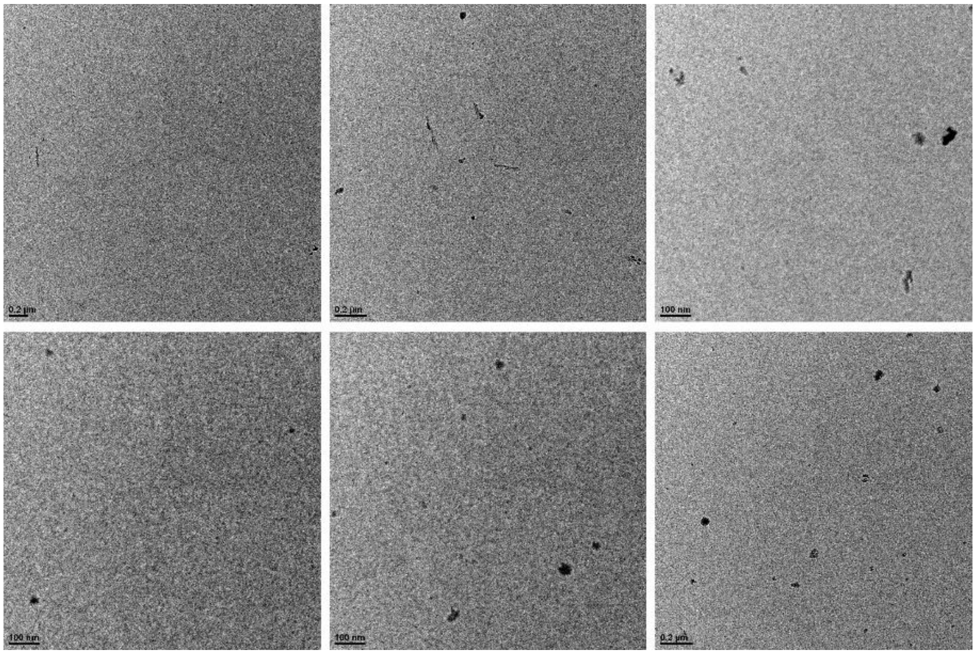

Figure S9. TEM imaging of α-synuclein (140 μM) with Ran.DNA (40 μM) (supernatant after centrifugation), from multiple positions on the TEM grid. Also see Figure 5B.

A

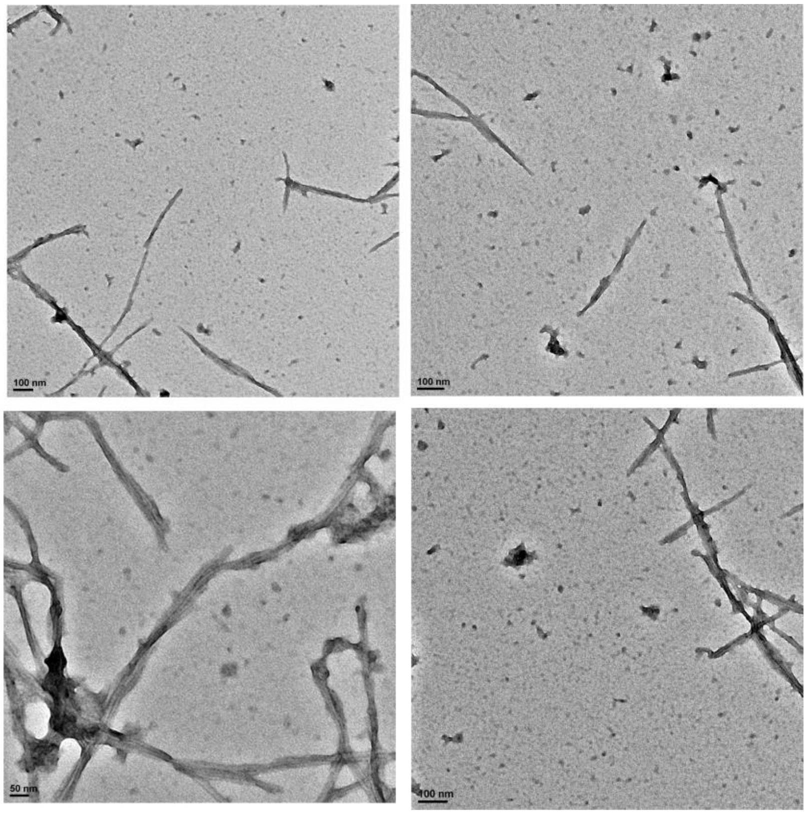

B

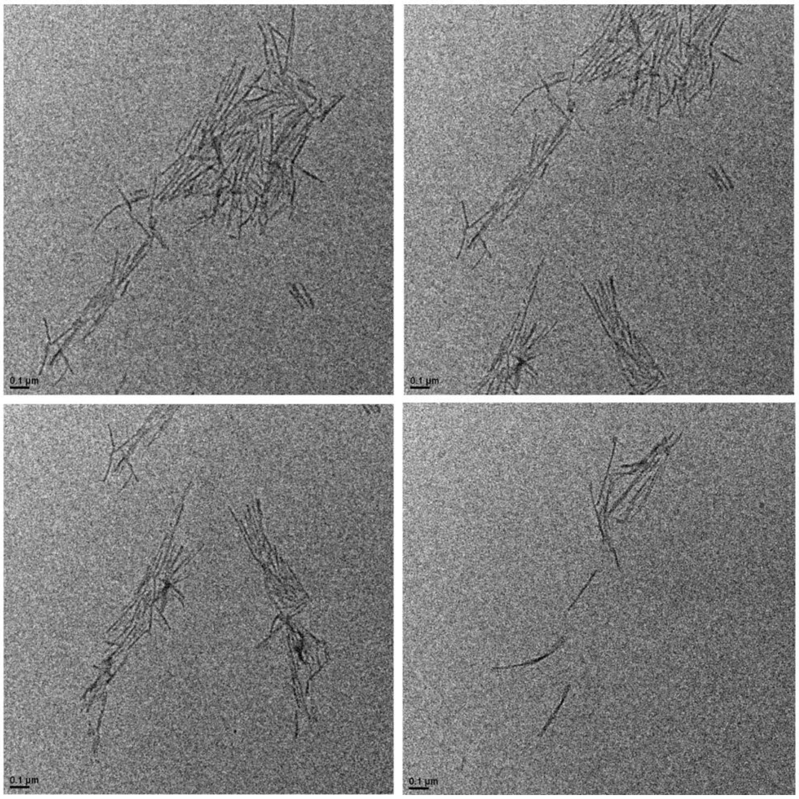

Figure S10. TEM imaging of α-synuclein (140 μM) with DNA (40 μM), from multiple positions on the TEM grid, without centrifugation to remove fibrils (whole solution). (A) α-synuclein with T-SO508; (B) α-synuclein with Ran.DNA.

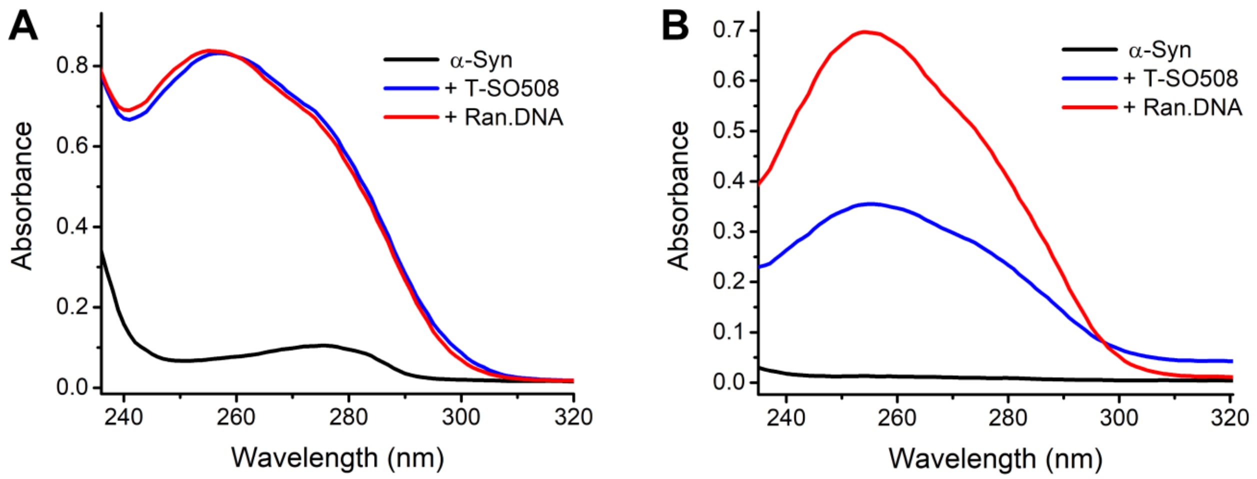

Figure S11. Absorbance spectrum of AUC sample before aggregation (A; whole solution) and after aggregation (B; supernatant after centrifugation to remove fibrils).

	Sample	$A_{260\ nm}$	$A_{276\ nm}$	$A_{260\ nm}/A_{276\ nm}$	p-value
**Before aggregation**	α-Syn	0.745 ± 0.004	1.345 ± 0.008		
	α-Syn + Ran.DNA	0.851 ± 0.112	0.655 ± 0.076	1.330 ± 0.020	0.167
	α-Syn + T-SO508	0.940 ± 0.226	0.732 ± 0.152	1.283 ± 0.039	
**After aggregation**	α-Syn	0.015 ± 0.003	0.011 ± 0.002		
	α-Syn + Ran.DNA	0.597 ± 0.018	0.419 ± 0.012	1.424 ± 0.003	<0.0001
	α-Syn + T-SO508	0.308 ± 0.014	0.225 ± 0.010	1.365 ± 0.001	

Table S2. Absorbance values of AUC samples before and after aggregation. Data are expressed as mean ± SD (n=3). Statistical significance comparing samples with Ran.DNA vs T-SO508 was computed using a non-parametric two-tailed *t*-test with Welch's correction. $p = 0.167$ ($t = 1.857$, df =2.98; before aggregation) and $p<0.0001$ ($t = 24.09$, df = 4.0; after aggregation). $p<0.05$ was considered significant. α-Syn = α-synuclein.

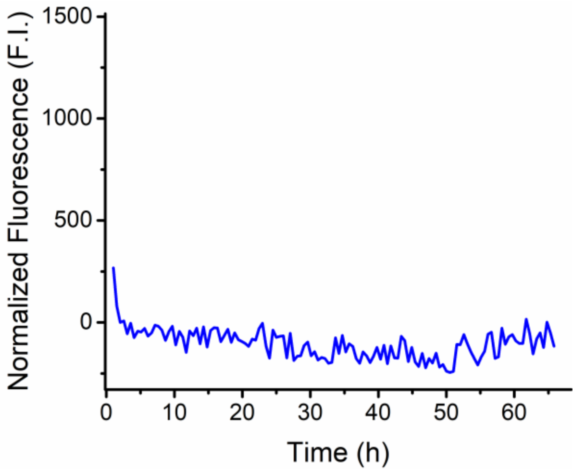

Figure S12. α-synuclein (70 μM) incubated in the presence of 60 μM ThT and 45 μM T-SO508.

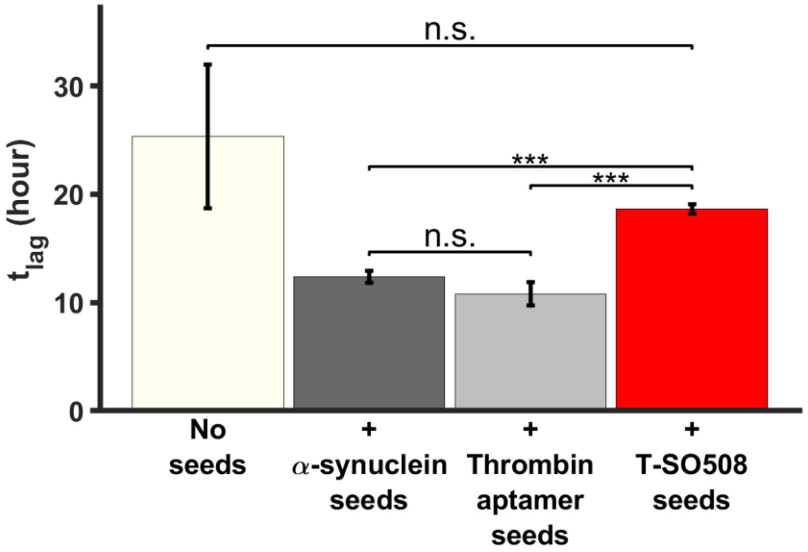

Figure S13. Experiment as shown in Figure 7, including comparison to a sample with no seeds added (white). Error bars are one standard error (n = 4; p-values for two-sample t-test are indicated: n.s. = not significant, *** indicates p<0.001).

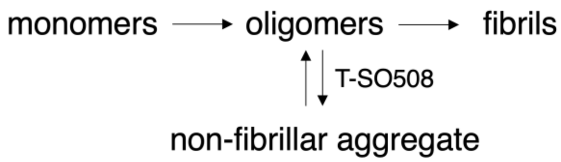

Figure S14. Suggested overall mechanism of T-SO508 effect on α-synuclein aggregation.
